# *Perna canaliculus* as an Ecological Material in the Removal of *o*-Cresol Pollutants from Soil

**DOI:** 10.3390/ma14216685

**Published:** 2021-11-05

**Authors:** Magdalena Zaborowska, Jadwiga Wyszkowska, Agata Borowik, Jan Kucharski

**Affiliations:** Department of Soil Science and Microbiology, University of Warmia and Mazury in Olsztyn, Plac Łódzki 3, 10727 Olsztyn, Poland; m.zaborowska@uwm.edu.pl (M.Z.); agata.borowik@uwm.edu.pl (A.B.); jan.kucharski@uwm.edu.pl (J.K.)

**Keywords:** *o*-cresol, biodiversity, soil enzymes, soil microbiome, biosorbent, *Perna canaliculus*

## Abstract

Soil contamination with cresol is a problem of the 21st century and poses a threat to soil microorganisms, humans, animals, and plants. The lack of precise data on the potential toxicity of *o*-cresol in soil microbiome and biochemical activity, as well as the search for effective remediation methods, inspired the aim of this study. Soil is subjected to four levels of contamination with *o*-cresol: 0, 0.1, 1, 10, and 50 mg *o*-cresol kg^−1^ dry matter (DM) of soil and the following are determined: the count of eight groups of microorganisms, colony development index (CD) and ecophysiological diversity index (EP) for organotrophic bacteria, actinobacteria and fungi, and the bacterial genetic diversity. Moreover, the responses of seven soil enzymes are investigated. *Perna canaliculus* is a recognized biosorbent of organic pollutants. Therefore, microbial biostimulation with *Perna canaliculus* shells is used to eliminate the negative effect of the phenolic compound on the soil microbiome. Fungi appears to be the microorganisms most sensitive to *o*-cresol, while *Pseudomonas* sp. is the least sensitive. In *o*-cresol-contaminated soils, the microbiome is represented mainly by the bacteria of the *Proteobacteria* and *Firmicutes* phyla. Acid phosphatase, alkaline phosphatase and urease can be regarded as sensitive indicators of soil disturbance. *Perna canaliculus* shells prove to be an effective biostimulator of soil under pressure with *o*-cresol.

## 1. Introduction

The European Commission (EC) and the United States Environmental Protection Agency (USEPA) have listed cresols as priority contaminants based on toxicity and environmental concerns [1]. Approximately 15 × 10^9^ kg of phenolic compounds are used annually worldwide [2]. The production capacity for cresol currently amounts to more than 3 × 10^9^ kg [3]. For this reason, *o*-cresol, a phenolic derivative belonging to volatile organic compounds, is widespread in many types of industrial wastewater, 14% of which penetrates into the soil [2,4]. *o*-Cresol is mainly released to the environment from steel foundries, leather and textile manufacturing, facilities producing epoxy resins for sealing integrated circuits (chips) or glass fibres, olive oil production, chemical paper-pulp and paper processing mills, and pharmaceutical plants [4,5,6,7,8]. *o*-Cresol is also used for the synthesis of medicines and synthetic antioxidants e.g., 3,5-Di-*tert*-butyl-4-hydroxytoluene (BHT) and butylated hydroxyanisole (BHA) as well as pigments [9]. Its sources in the soil also include selective pesticides, inter alia dichloro-diethyl-trichloroethane, 2,4-dichlorophenoxyacetic acid and pentachlorophenol, degraded by plants and fungi as well as e-waste, such as waste electrical or electronic equipment. According to the United Nations University, the generation of e-waste will reach 50 × 10^9^ kg by the year 2020 [10,11]. Air, surface and ground waters and soils become contaminated with phenolic compounds as a result of anthropogenic operations, such as waste incineration and wood-burning [12]. Significant cresol sources of great concern include flotation processes in mining and metallurgical industries, particularly copper flotation, oil refining and coal conversion [3,6]. The refining of crude oil is a cause of concern, as the Organization of the Petroleum Exporting Countries predicts the demand for oil in the years 2015–2040, in order to increase by 16.4 million barrels per day [13]. Cresol toxicity in the soil is affected by indirect metabolites generated by the splitting of phenolic compounds, e.g., acyl halides or hydroquinone [14,15].

**Figure 1:**
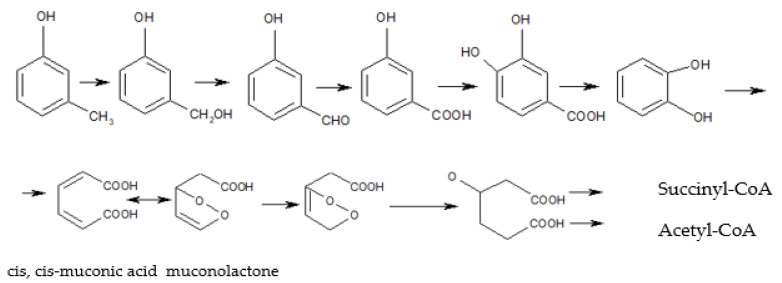
Cresol biodegradation pathway along the ortho (catechol) pathway [23].

In the natural environment, cresols are formed during lignin and tannin biodegradation [16]. Many phenolic root exudates, including phenolic acids, flavonols, or flavonoids such as procyanidins (PACs), consist of catechin and/or epicatechin units linked by interclavulanic bonds. They have been identified as antimicrobial compounds.They inhibit the activity of gram-negative and gram-positive bacteria, or stimulate the growth of subpopulation-degrading aromatic compounds [17,18]. However, it appears more important to understand the mechanism of catabolic route evolution, which also offers an opportunity to isolate further bacterial strains. Microorganisms are indirectly involved in many catabolic biochemical reactions that initiate this process [19]. The enzymes catalysing these reactions include monooxygenases, such as phenol hydroxylase (MHS) (EC 1.14.13.7) performing the aromatic ring hydroxylation [20], inducible enzymes, derivatives of the cytochrome P-450 with iron ion at various oxidation degrees in the active centre [21] and toluene/o-xylene monooxygenase (ToMO), discovered in the *Pseudomonas stutzeri* OX1 genome. The key enzymes in the aerobic biodegradation pathway also include dioxygenases, catechol 1,2-dioxygenase or catechol 2,3-dioxygenase, and extradiol enzymes with non-haem Fe(II), thanks to which, phenolic compounds with a methyl group are meta-split, both distally and proximally, to the intermediate products of the Krebs cycle metabolism, such as pyruvate, succinate and acetyl-coenzyme A [22]. An exemplary pathway for oxygen degradation of cresols is presented in Figure 1.

Denitrification microorganisms that reduce sulphates are responsible for the anaerobic catabolism of cresol [24]. An important initiating enzyme is *p*-cresol methylhydroxylase (PCMH) (WE.1.17.99.1), which has been previously isolated from *Geobacter metallireducens* [25].

The key property that controls the concentration, mobility, toxicity and fate of cresols in the environment is the sorption of these compounds on soils, which is a process determined by the presence of organic matter (OM) [26]. The hydrophobic effect, hydrogen bonds and electrostatic interactions are involved in sorption [27]. The solvent effects occur when some adsorption sites for phenol are occupied by water molecules. Water molecules can be adsorbed on the surface oxygen groups by hydrogen bonds. As a result, water molecules in solvent effects decrease the adsorption capacity of phenol by a hydrogen bond [28]. The influence of soil moisture and van der Waals forces for physisorption and chemical reactions for chemisorption should also not be underestimated [29]. Moisture affects the solubility and, thus, the availability of phenolic compounds and soil pH. However, cresols can be removed within a wide pH range from 2.0 to 11.0 [30,31]. According to Fu et al. [28], the adsorption of phenol onto the activated biocharts was predominantly controlled by the chemisorption. Furthermore, the functional groups on its outer surfaces can attract the phenol molecules to the inner surfaces through the “π-π dispersion interaction” and the “donor-acceptor effect”. Both the mechanisms of cresol toxicity to microorganisms and the response of soil microbiomes to cresol soil contamination are limited [32].

There are, however, opportunities to address soil cresol contamination using already manufactured waste products. China is the largest producer of sea bivalve mussels, accounting for 84% of global production [33]. Spain is also one of the most important producers of mussel in the world [34]. Most of this mussel production is processed by the cannery industry and generates vast amounts of waste, up to 65,682 tonnes per year, which are mainly shells. Creating value for waste shells will relieve strain on landfills and aligns with the goals of a circular economy. Thus, the application of mussel shell as a biostimulant could address the above environmental issue. Mussel shell powder is often used to treat sewage or to improve soil quality. In terms of their composition, the main component of mussel shells is calcium carbonate (CaCO_3_). *Perna canaliculus* shells also contain a small amount of organic matter, such as protein and carbohydrates [35]. Additionally, *Perna* sp. mussels present all the desirable characteristics for biomonitoring applications. In this regard, *Perna* sp. has been proposed as a bio-indicator to estimate the contamination degree and effects of pollutants on biota [36,37]. These aquatic organisms contain a wide variety of enzymes, e.g., cytochrome P450 (CYP450) enzymes and transferase enzymes, including glutathione S-transferase (GST), responsible for detoxifying toxic compounds [38]. It should also be emphasized that the potential of mussels to eliminate the negative effects of phenolic compounds, including *o*-cresol, has not been studied on a larger scale so far. Instead, the retention of sulphonamides, tetracyclines and the adsorption and desorption ability of mussel shells toward heavy metals in the soil were mainly determined [39,40].

Moreover, although the awareness of the varied degradation activity of microorganisms is increasing, it is not correlated with knowledge about the sensitivity of soil enzymes. Soil enzymes are recognised as early indicators of changes in the intensity of biological processes, and, importantly, the scale of soil degradation [41,42,43]. The main research hypothesis relates to the inhibitory effect of *o*-cresol on the biochemical and microbiological activity of the soil, also inducing changes in soil biodiversity. Therefore, the study aimed to determine the effect of *o*-cresol on both soil biochemical activity and the response of soil microorganisms, including their count and biodiversity. It is also important to determine the scale of the hypothetically beneficial effect of an innovative substance (*Perna canaliculus* mussel meal) on the soil condition. 

## 2. Materials and Methods

### 2.1. Experimental Design

Experimental soil, consisting of a topsoil layer at a depth of 0–20 cm (classified as Eutric Cambisol), was collected from the Educational and Experimental Station in Tomaszkowo (NE Poland, 53.7161′ N, 20.4167′ E). This agricultural area has been developed for varietal crop breeding as well as conducting crop rotation experiments. Located on the East-European Plain, within the East Baltic-Belarusian Lowlands region, the facility covers a total area of 4.5 ha (inclusive of buffer strips). During the growing season, when soil samples were taken for the experiment, the soil was sown with *Avena sativa* cv. *Bingo* (oats). Before the experiment, the soil was classified as loamy sand (sand—69%, silt—28%, and clay—3%), as determined in accordance with the international system of soil classification [44]. Soil physico-chemical properties were determined as per Borowik et al. [45] and are presented in Table 1. 

Because the response of soil enzymes and the soil microbiome has so far been studied to a negligible extent, this study is conducted as a greenhouse pot experiment under monitored conditions to reduce confounding variables. The length of the day ranged from 14 h 4 min to 16 h 30 min, the average air temperature was 16.5 °C ± 10 and average air humidity was 77.5% ± 6.8. The pot experiment was set up with five replicates. Thus, the experiment was carried out in 150 pots based on the following main variable factors: (1) 5 levels of *o*-cresol doses: 0; 0.1; 1; 10 and 50 mg of *o*-cresol kg^−1^ DM of soil, (2) 2 levels of *Perna canaliculus* mussel meal addition: 0; 5 mg kg^−1^ DM of soil, and (3) 3 experiment durations: 15, 30 and 45 days. After mixing with *o*-cresol and *Perna canaliculus* mussel meal, 1 kg of the soil was put into each 1.5 dm^3^ pot. On the day of setting up the experiment, the soil was brought to the level of 60% water-holding capacity. Soil moisture was maintained gravimetrically every 24 h.

### 2.2. Characteristics and Determination of o-Cresol Residues in the Soil

The subject of the study was *o*-cresol, one of three structural isomers of cresol, which has its methyl substituent in the ortho-position [51]. According to the Sigma Aldrich safety data sheet, *o*-cresol has the form of a colourless liquid with a phenolic smell and purity ≥ 98.0% (HPLC). Selected chemical and physical properties are presented in Table 2 and Table 3 [51].

*O*-Cresol dissolves well in ethanol, ethyl ether and chloroform, and was therefore added to the soil as a 3:1 ratio ethanol, known as *o*-cresol solution. Ethanol was rapidly degraded in soil as the degradation time is from three hours to two days after application [52]. In the research, *o*-cresol dissolved in ethanol was added in the amount of 5 cm^3^ 1 kg^−1^ DM of soil. Ethanol was also added to the control pots. The level of soil contamination with *o*-cresol was determined based on the permissible cresol concentration of 0.1 mg kg^−1^ of soil in soils classified as agricultural land at a depth of 0–0.3 m, parallel to the research assumptions [53]. The fact that cresols have been included in the ATSDR’s Substances Priority List [54], which consequently means that they are characterised by a high frequency of occurrence in the environment and toxicity, was also taken into account. 

The determination of *o*-cresol in the soil was conducted using the gas chromatography–mass spectrometry (GC–MS) method according to the research procedure PB-218/LF. Sample preparation involved the extraction of analytes with acetonitrile in an acidic environment, followed by derivatisation with acetic anhydride. 

### 2.3. Characteristics of Perna Canaliculus Mussel Meal

*Perna canaliculus*, a green-lipped mussel, is an endemic New Zealand species exported to approximately 60 countries, mainly to the USA, China and Thailand. Its production increased from 7 tonnes in 1971 to 85,857 Mg in 2018 [55]. The study used New Zealand *Perna canaliculus* mussel shell meal. *Perna canaliculus* are recognised as an indicator species for ecosystems contaminated with heavy and radioactive metals [56,57]. The nitrate nitrogen and ammonium nitrogen contents were 5 mg kg^−1^ DM NO_3_^−^-N and 1902 mg kg^−1^ DM NH_4_^+^-N. *Perna canaliculus* shells were used as a substance eliminating the negative effects of *o*-cresol. The dose of mussel shells was determined based on preliminary studies and literature data [39,40,58,59]. Our previous research [58] showed that *Perna canaliculus* shells used in the dose of 5 mg kg^−1^ DM are a very good biostimulator of both biochemical and microbiological activity.

### 2.4. Determination of Soil Microorganisms 

In each soil sample collected on days 15, 30 and 45 of the study, the count was determined for five microbial groups, i.e., ammonifying bacteria (Am), nitrogen-immobilising bacteria (Im), *Arthrobacter* sp., *Pseudomonas* sp. and *Azotobacter* sp. The counts of organotrophic bacteria (Org), actinobacteria (Act) and fungi (Fun) were also determined, which became the matrix for determining two indices: the colony development (CD) index and the eco-physiological diversity (EP) index, described by the following formulas:

(1) $$\mathrm{CD}=[\frac{N_{1}}{1}+\frac{N_{2}}{2}+\frac{N_{3}}{3}\ldots \ldots .\frac{N10}{10}]\text{·}100$$

where: $\frac{N1}{1},\frac{N2}{2},\frac{N3}{3},\ldots \;\frac{N10}{10}$ r—respective ratios of microbial colony numbers identified on each subsequent day of the experiment (*N*_1_, *N*_2_, *N*_3_, … *N*_10_) and:

EP = −Σ(pi · log pi)(2)

where: pi denotes the number of microbial colonies replicated on a specific day (*N*_1_, *N*_2_, *N*_3_… *N*_10_) divided by the number of all the colonies identified in the experiment (*N*_10_). 

The assays were conducted using the serial dilution method, with three replications. The microorganisms were cultured on Petri dishes, and the multiplying colony-forming units (cfu) were counted for the next ten days using a colony counter. The composition of microbiological mediums and the method for conducting the analysis was described by Borowik et al. [45]. All the microorganism groups were incubated at a constant temperature of 28 °C.

### 2.5. DNA Isolation and Bioinformatic Analysis of Bacterial Taxa

The application of mutanolysin and lysozyme in the Genomic Mini AX Bacteria +” kit (A&A Biotechnology, s. c., Gdańsk, Poland) ensured the effective extraction and precipitation of the genomic DNA, resulting from the digestion of cell walls of the bacteria particularly resistant to the lysis that preceded the determination of DNA. The mechanical lysis was conducted using a FastPrep-24 apparatus (MP Biomedicals LLC, Solon, OH, USA). The next stage included an additional purification using an Anti-Inhibitor Kit (A&A Biotechnology s. c., Gdańsk, Poland). Bacterial DNA was determined in the samples by the colorimetric method and confirmed using Real-Time PCR (A&A Biotechnology s. c., Gdańsk, Poland). Based on universal starters of the PCR mixture containing 1055F in the presence of SYBR pigment, the sequencing of the gene encoding the amplicon 16S sequences was conducted, based on the V3-V4 hypervariable region. The bio-informatic analysis was conducted by Genomed SA (Warsaw, Poland) using a MiSeq v2 Illumina sequencer (Illumina, Inc., San Diego, CA, USA) by assigning operational taxonomic units (OUT) to the sequencing reads on the 16S RNA gene amplicon sequencing in accordance with the taxonomic affiliation to the genus level. 

### 2.6. Determination of Soil Enzyme Activity

Biochemical analyses of soil samples were performed in three replications, at 15-day intervals (on days 15, 30 and 45 of the experiment). The activity of seven enzymes was determined: dehydrogenases (Deh) (EC 1.1), alkaline phosphatase (Pal) (EC 3.1.3.1), acid phosphatase (Pac) (EC 3.1.3.2), urease (Ure) (EC 3.5.1.5), arylsulfatase (Aryl) (EC 3.1.6.1), *β*-glucosidase (Glu) (EC 3.2.1.21) and catalase (Cat) (EC 1.11.1.6). The following substrates were used in the determination of enzyme activity: Deh-2,3,5-triphenyl tetrazolium chloride (TTC) [60], Cat-H_2_O_2_ (aqueous solution), Pal and Pac -disodium4-nitrophenyl phosphate hexahydrate (PNP), Ure–Urea (aqueous solution), Glu-4-nitrophenyl-*β*-D-glucopyranoside (PNG) and Aryl-potassium-4-nitrophenylsulfate (PNS) [61]. The units used to express the values of biochemical indicators and the detailed analysis procedures were described by Borowik et al. [45].

### 2.7. Statistical Data Analysis and Methodology of Calculations

The obtained study results were subjected to a statistical analysis using the Statistica 13.1 package [62]. Tukey’s test (HSD) was applied (at *p* = 0.01) to determine the homogeneous variances between soil enzymes and groups of microorganisms subjected to the pressure of increasing levels of soil contamination with *o*-cresol on days 15, 30 and 45 of the experiment. Multidimensional PCA analysis was employed to determine the degree of soil biostimulation with the New Zealand mussel *Perna canaliculus*. The response of soil enzymes and particular microbial groups in both the control soil and the soil contaminated with *o*-cresol was assumed to be the matrix. The changing trends are presented using the impact factor of a biostimulating substance, as described with the following formula:

(3) $${\mathrm{IF}}_{\mathrm{Pc}}=\frac{A_{\mathrm{Pc}}}{A_{C}}$$

where: IF_Pc_—the factor of the impact of New Zealand mussel *Perna canaliculus* (Pc), (IF_Pc_ < 1—inhibition of the enzyme activity and groups of microorganisms by *Perna canaliculus*; IF_Pc_ > 1—stimulation of the soil enzyme activity and groups of microorganisms by *Perna canaliculus*; A_Pc_—enzyme activity and groups of microorganisms in the soil subjected to the increasing *o*-cresol contamination pressure and further subjected to biostimulation with *Perna canaliculus*, A_C_—enzyme activity and groups of microorganisms in the control soil and non-contaminated with *o*-cresol subjected to biostimulation with *Perna canaliculus.*

The metagenomic data are presented graphically with the number of OTU lower than 1% in relation to the total number of OTU having been eliminated. This pool of results was statistically analysed using a Circos 0.68 package, which presents data in a circular array [63], a gplots library [64], a thermal map generated based on the RStudio v1.2.5033 software [65] and the R v3.6.2 system [66]. An analysis of statistical significance, employed to trace the changes in biodiversity of the soil microbiome, is the bilateral statistical hypothesis test, G-test (w/’Yates’) + Fisher’s, including the Asymptotic with CC confidence interval method [67], configured using STAMP 2.1.3 software. 

## 3. Results

### 3.1. Counts and Diversity of Microorganisms

**Figure 2:**
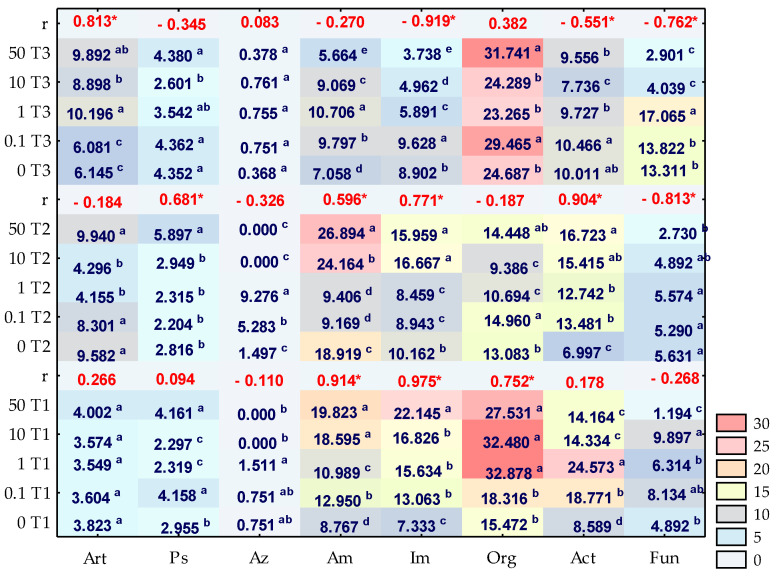
Number of microorganisms in soil contaminated with *o*-cresol on day 15, 30 and 45 of the experiment (cfu 10^n^ kg^−1^ DM of soil); Org—organotrophic bacteria (10^9^), Act—actinomycetes (10^9^), F—mold fungi (10^7^), Ps—*Pseudomonas* sp. (10^8^), Art—*Arthrobacter* sp. (10^8^), Im—nitrogen-immobilising bacteria (10^8^), Am—ammonifying bacteria (10^8^) and Az—*Azotobacter* sp. (10^3^). Homogeneous groups are specified in columns, for each group of microorganisms, depending on the increasing doses of *o*-cresol on T1—15 day, T2—30 day, T3—45 day of the experiment are denoted with letters (a–e), r—Pearson’s linear correlation coefficient, * significant for *p* = 0.05, *n* = 14.

The soil microbiome response to the contamination of soil, with *o*-cresol significantly corresponded with the duration of the exposure to this phenolic compound (Figure 2). On day 15 of the experiment, *o*-cresol applied into the soil at a level of 50 mg kg^−1^ DM of soil moderated the development of microorganisms, while contributing to an almost two-fold increase in the count of organotrophic bacteria, ammonifying bacteria and actinobacteria, and an almost three-fold increase in the count of nitrogen-immobilising bacteria. The presence of *o*-cresol also increased the count of *Pseudomonas* sp. by 14.07% in relation to the control pots, whereas the lowest contamination level of 0.1 mg *o*-cresol kg^−1^ DM of soil triggered a similar response in this group of microorganisms. The pressure of 50 mg of *o*-cresol kg^−1^ DM of soil resulted in a 76% reduction in the fungal count compared to the control sample. Similar trends were observed on day 30 of the study, yet the impact of this phenolic compound decreased. A stimulation of the count of actinomycetes and *Pseudomonas* sp. was observed. However, with regard to ammonifying bacteria and nitrogen-immobilising bacteria, a slightly lower increase in their count, (by 42% and 57%, respectively) was noted. Negative interference of *o*-cresol with the multiplication of fungi was also observed. Their count decreased by 51%, as compared to the control object. The trends obtained after 45 days of the experiment appeared to be interesting. They include to the inhibition of microorganism exposed to the effect of 50 mg *o*-cresol kg^−1^ DM of soil, a 78% decrease in fungal count, as well as a decrease in nitrogen-immobilising bacterial count by 58%, ammonifying bacteria count by 19% and actinobacterial count by 4%. However, they also reflect the positive effect of the phenolic compound on the count of *Arthrobacter* sp. and organotrophic bacteria (which increased by 61% and 29%, respectively). 

**Figure 3:**
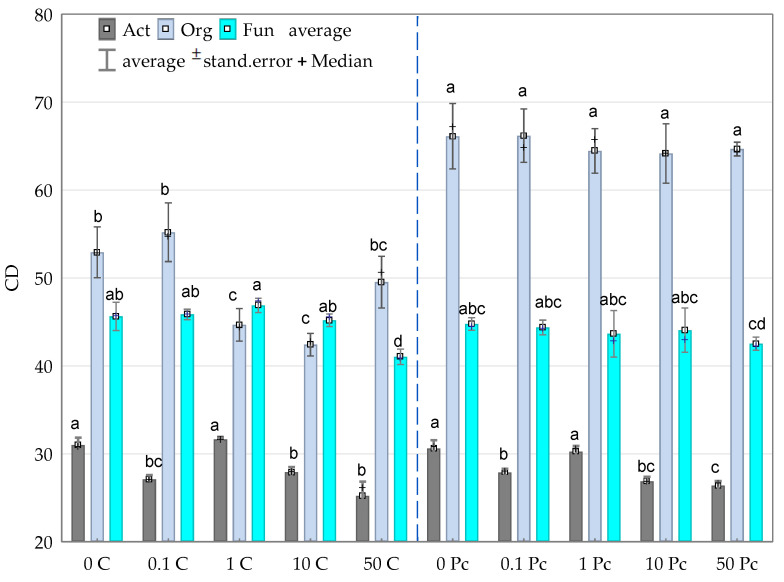
Colony development index (CD) for Org—organotrophic bacteria, Act—actinomycetes and Fun—fungi in soil contaminated with increasing doses of *o*-cresol during 15, 30 and 45 days of the experiment (average value); Homogeneous groups denoted with letters (a–d) were calculated separately for each group of microorganisms; 0; 0.1; 1; 10; 50—doses of *o*-cresol (mg kg^−1^ DM of soil); C—control; and Pc—*Perna canaliculus* (for abbreviations see Figure 2).

**Figure 4:**
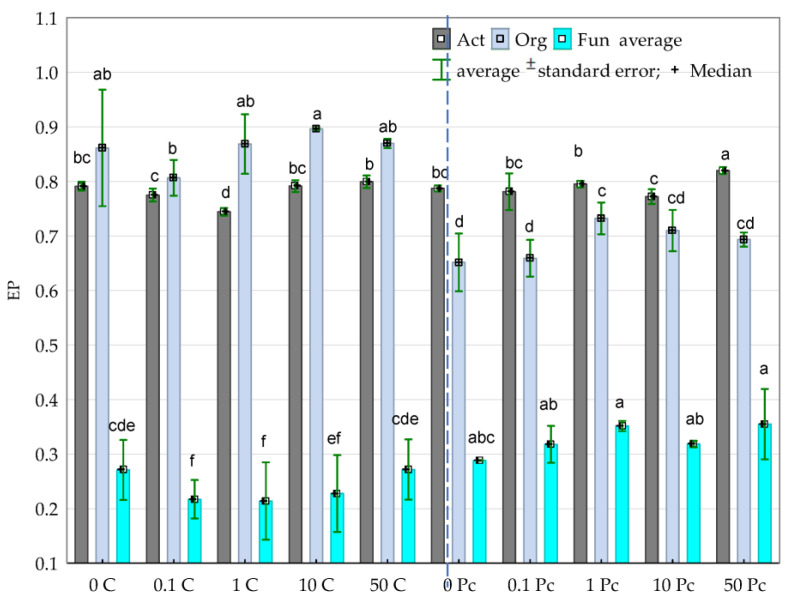
Ecophysiological diversity factor (EP) for Org—organotrophic bacteria, Act—actinomycetes and Fun—fungi in soil contaminated with increasing doses of *o*-cresol during 15, 30 and 45 days of the experiment (average value); Homogeneous groups denoted with letters (a–f) were calculated separately for each group of microorganisms, 0; 0.1; 1; 10; 50—doses of *o*-cresol (mg kg^−1^ DM of soil); C—control; Pc—*Perna canaliculus* (for abbreviations see Figure 2).

The obtained high CD values for organotrophic bacteria and fungi indicate the dominance of rapidly growing microorganisms among these groups. In turn, actinobacteria were classified as so-called “slowly-growing” microorganisms, irrespective of the level of soil contamination with *o*-cresol (Figure 3). The application of the phenolic compound into the soil did not contribute to accelerating the rate of organotrophic bacteria development but actually inhibited this process, as opposed to the biostimulation with the *Perna canaliculus* mussel shells. This phenomenon was emphasized by the obtained homogeneous groups. Based on these groups, the negative effect of the compilation of increasing levels of soil contamination with *o*-cresol and a biostimulating substance on the ecophysiological diversity (EP) index, assigned to these microbial groups, was observed (Figure 4). The applied *Perna canaliculus* mussel had no stimulating effect on the rate of fungal multiplication, yet it increased fungal ecophysiological diversity, particularly in the pots contaminated with *o*-cresol. 

**Figure 5:**
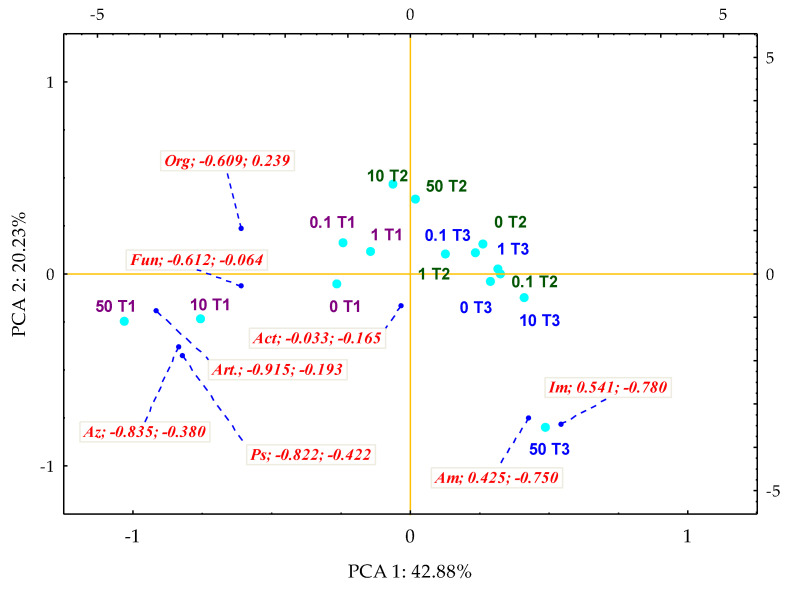
Coefficients of impact (IF_Pc_) of *Perna canaliculus* on the number of microorganisms in soil contamination with *o*-cresol—PCA method; the end of the vector of the primary variable: Org—organotrophic bacteria, Act—actinomycetes, F—mold fungi, Ps—*Pseudomonas* sp., Art.—*Arthrobacter* sp., Im—nitrogen immobilising bacteria, Am—ammonifying bacteria, Az—*Azotobacter* sp.; cases: doses of *o*-cresol mg kg^−1^ DM of soil: 0; 0.1; 1; 10; 50; time: T1—day 15, T2—day 30, T3—day 45 of the experiment.

The obtained results correspond to the conducted multidimensional PCA analysis, which illustrates the differences in the effect of *Perna canaliculus* on the microbiological activity of the soil. Its effectiveness in stimulating this parameter was traced using the IF_Pc_ factor (Figure 5). It was found that the first principal component (PCA1), which explains 43% of the total data variance, generated negative values of the vectors of primary variables for fungi (−0.612), similar to most of the analysed bacterial groups, except for nitrogen-immobilising bacteria (0.541) and ammonifying bacteria (0.425). The dislocation of the cases indicated that on the 15 day of exposure to 10 and 50 mg *o*-cresol kg^−1^ DM of the soil, the mussel shells had a positive effect on the number of *Azotobacter* sp., *Pseudomonas* sp. and *Arthrobacter* sp. A spectacularly positive result was also achieved for nitrogen-immobilising bacteria and ammonifying bacteria in pots with the highest *o*-cresol contamination level, on day 45 of the experiment.

**Figure 6:**
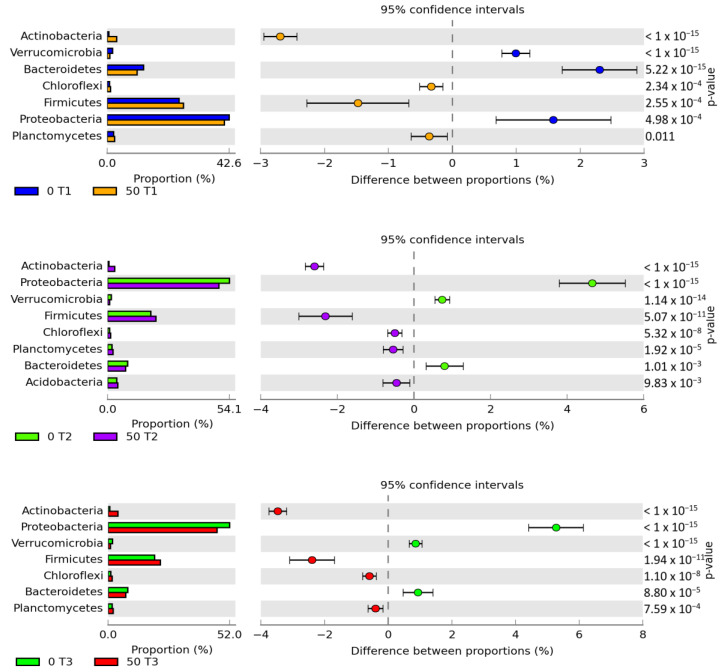
Relative abundance of the dominant bacterial phylum in soil with a difference between proportions ≥1%. A total of 0; 50—doses of *o*-cresol (mg kg^−1^ DM of soil); T1—day 15, T2—day 30, T3—day 45 of the experiment.

Of the distinguished phyla, the applied phenolic compound generated the greatest and most varied abundance of OTU for proteobacteria (Figure 6). In control pots, the obtained abundance was as follows: day 15–10,500 OTU, day 30–15,585 OTU and day 45–14,666 OTU. Following the application into the soil of 50 mg *o*-cresol kg^−1^ DM of soil, the OTU count decreased by 2%, 5% and 5%, respectively. The second most important phylum determining the soil microbiome activity was *Firmicutes*, which accounted for 22% of the population, irrespective of the study duration. Nevertheless, on days 30 and 45, 50 mg *o*-cresol kg^−1^ DM of soil decreased the count of *Firmicutes* OTUs by 5% and 4% respectively, in relation to the OTU value (6059) obtained on day 15 of the experiment.

**Figure 7:**
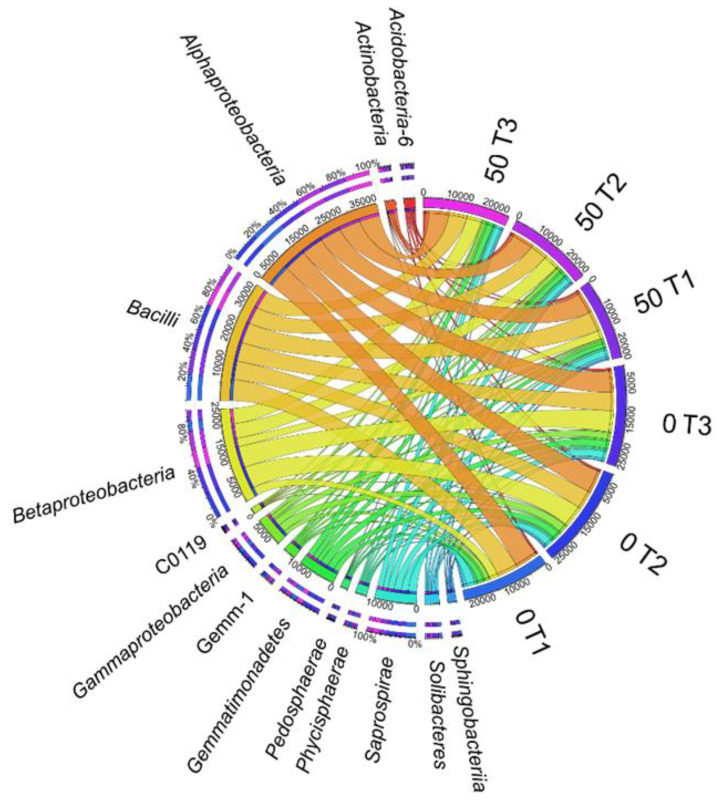
The relative abundance of dominant classes of bacteria in soil with a difference between proportions ≥1%. 0; 50—doses of *o*-cresol (mg kg^−1^ DM of soil); T1—15th day, T2—30th day, T3—45th day of research.

Within the lower taxonomic units, five of the most numerous classes were distinguished, i.e., *Alphaproteobacteria*, *Bacilli*, *Betaproteobacteria*, *Saprospirae* and *Gemmatimonadetes*, with a significant dominance of *Alphaproteobacteria* and *Bacilli* assigned, respectively, to the *Proteobacteria* and *Firmicutes* phyla (Figure 7). Irrespective of the study duration, *o*-cresol reduced the OTU for the classes *Betaproteobacteria*, *Saprospirae* and *Gemmatimonadetes* and increased this value for *Alphaproteobacteria* and *Bacilli*. It should be emphasised that the intensity of the inhibitory effect of the phenolic compound on the OTU count was the highest on day 45 of the experiment. 

**Figure 8:**
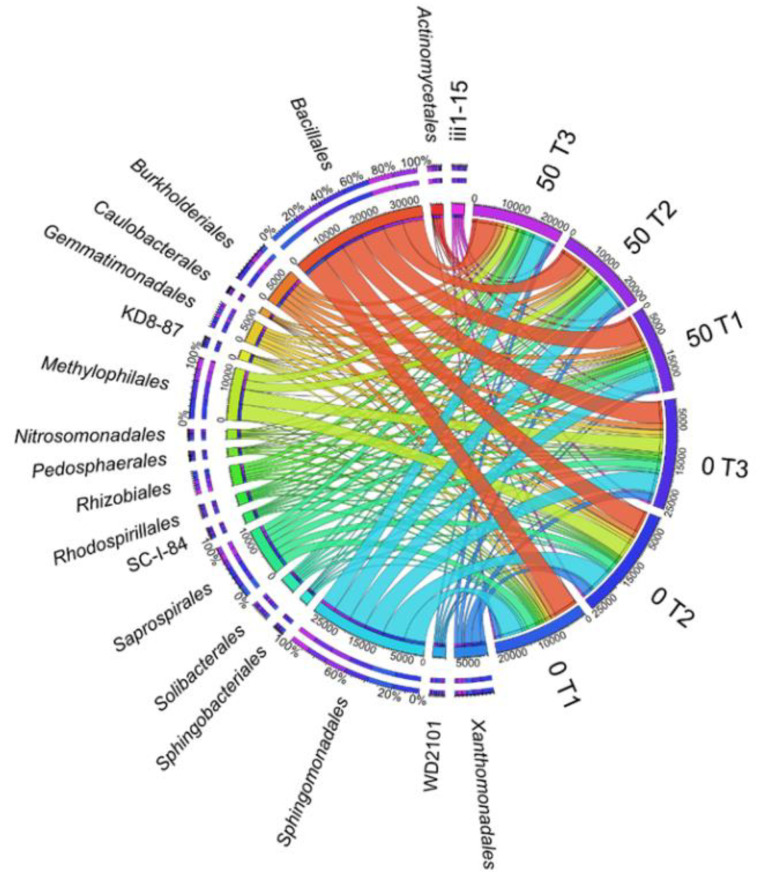
The relative abundance of dominant orders of bacteria in soil with a difference between proportions ≥1%. 0; 50—doses of *o*-cresol (mg kg^−1^ DM of soil); T1—15th day, T2—30th day, T3—45th day of research.

According to the OTU values determined at the order level, the main dominant *Bacillales* taxon, belonging to the *Firmicutes* phylum, was selected and its abundance increased after exposure to 50 mg *o*-cresol kg^−1^ DM of soil, irrespective of its duration (Figure 8). The *Bacteroidetes* phylum was represented by the *Saprospirales* order, while for the *Proteobacteria* phylum, bacteria of the *Sphingomonadales*, *Burkholderiales,* and *Xanthomonadales* orders were dominant, whereas bacteria of the *Xanthomonadales* genus appeared to be particularly sensitive to *o*-cresol applied to the soil. 

**Figure 9:**
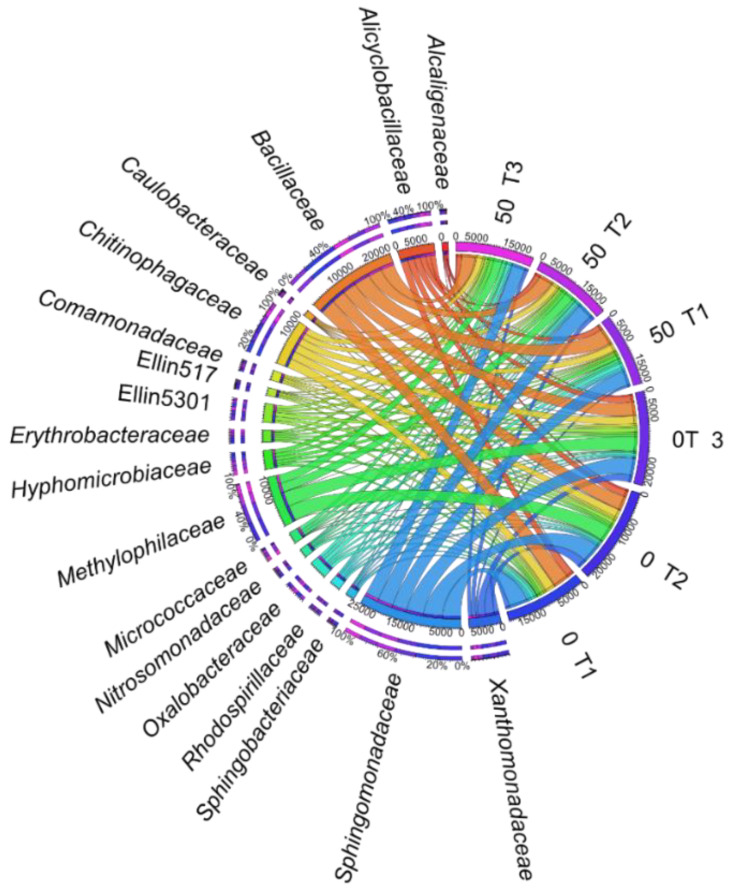
The relative abundance of dominant family of bacteria in soil with a difference between proportions ≥1%. 0; 50—doses of *o*-cresol (mg kg^−1^ DM of soil); T1—15th day, T2—30th day, T3—45th day of research.

Its effect at the order level was reflected in the lower taxonomic unit (family), in which two taxons most represented by bacteria, i.e., *Sphingomonadaceae* and *Bacillaceae* (Figure 9) were identified. The highest OTU values in contaminated objects were noted for *Sphingomonadaceae* on day 30 (4079 OTU) and for *Bacillaceae* on day 15 of the experiment (4313 OTU). 

**Figure 10:**
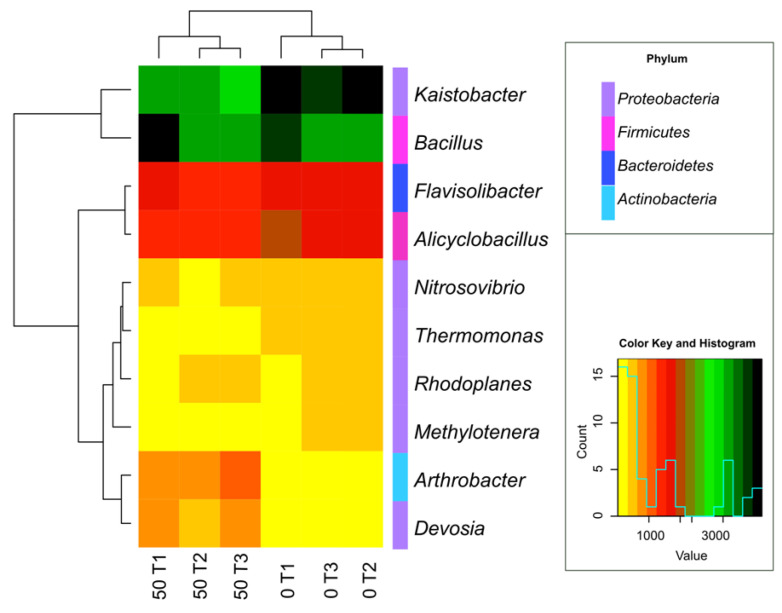
Heat map and associations of the number of bacterial genus in soil with a difference between proportions ≥1%. 0; 50—doses of o-cresol (mg kg^−1^ DM of soil); T1—15th day, T2—30th day, T3—45th day of the experiment.

**Figure 11:**
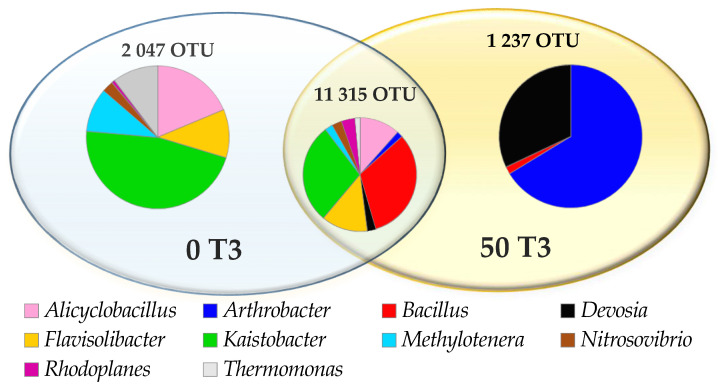
Read of the common operational taxonomic units (OTU) of bacteria ≥1% genus level in the control soil (0 T3) and in soil contaminated with 50 mg *o*-cresol (50 T3) on day 45 of the experiment.

The study also highlighted the bacterial genera (Figure 10 and Figure 11), the most numerous of which include *Kaistobacter*, belonging to the *Proteobacteria* phylum, and *Bacillus* and *Alicyclobacillus*, belonging to the phylum *Firmicutes* and *Flavisolibacter* assigned to the *Bacteroidetes* phylum. Ten genera common for all objects were identified. However, the special abundance of OTU in the soil under the 45-day *o*-cresol pressure for *Arthrobacter, Devosia* and *Bacillus* genus, belonging to phyla *Actinobacteria*, *Proteobacteria* and *Firmicutes*, respectively, should be emphasized (Figure 10).

### 3.2. Enzyme Activity 

**Figure 12:**
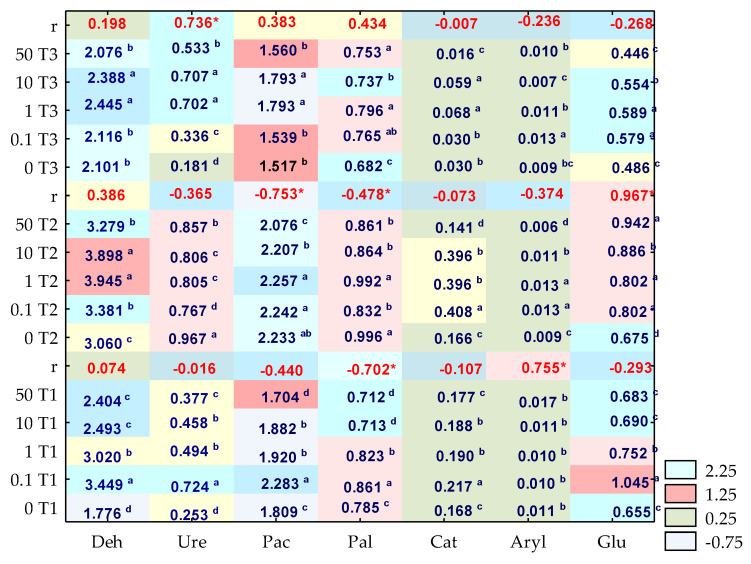
Enzymatic activity in soil contaminated with o-cresol on day 15, 30 and 45 of the experiment, (kg^−1^ DM of soil h^−1^); Deh—dehydrogenases (μMol TFF); Cat—catalase (Mol O_2_); Ure—urease (mMol N-NH_4_); Pal—alkaline phosphatase; Pac—acid phosphatase; Glu—β-glucosidase; Aryl—arylsulfatase (mMol 4-nitrofenol PN). Homogeneous groups are specified in columns for each enzyme, depending on the increasing doses of *o*-cresol during T1—15 days, T2—30 days, T3—45 days of the experiment and denoted with letters (a–d), r—Pearson’s linear correlation coefficient, * significant for *p* = 0.05, *n* = 14.

The activity of enzymes in the soil contaminated with *o*-cresol was significantly moderated by both the xenobiotic applied into the soil and the duration of the experiment (Figure 12). The disturbance of the biochemical balance was noted on days 15, 30 and 45 of the study. On day 15, it was mainly due to the inhibition of *o*-cresol in relation to Pac and Pal. The activity of these enzymes decreased by 6.16% and 10%, respectively, in the objects exposed to the effects of 50 mg *o*-cresol kg^−1^ DM of soil. Moreover, the formed homogeneous groups indicate an unprecedented, significant increase in the Ure activity by 65% compared to the control objects, following the application into the soil of 0.1 mg *o*-cresol kg^−1^ DM of soil. Interestingly, in a parallel object, on day 30 of the experiment, the studied phenolic compound contributed to the 13% inhibition of this enzyme’s activity. Another fact worth emphasising is the significant stimulation of Glu (r = 0.967), correlated with increasing soil contamination with *o*-cresol. The biotic stress caused by the contamination of soil with this phenolic compound for 45 days generated the following sequence of sensitivity of individual enzymes: Glu > Aryl > Cat > Deh > Pac > Pal > Ure. In this pool of objects, soil contamination with *o*-cresol at a level of 1 mg kg^−1^ DM of soil contributed to an increase in the activity of all enzymes compared to control samples. A particularly positive increase (almost four-fold) was noted for Ure, while a 2.5-fold increase was observed for Cat.

**Figure 13:**
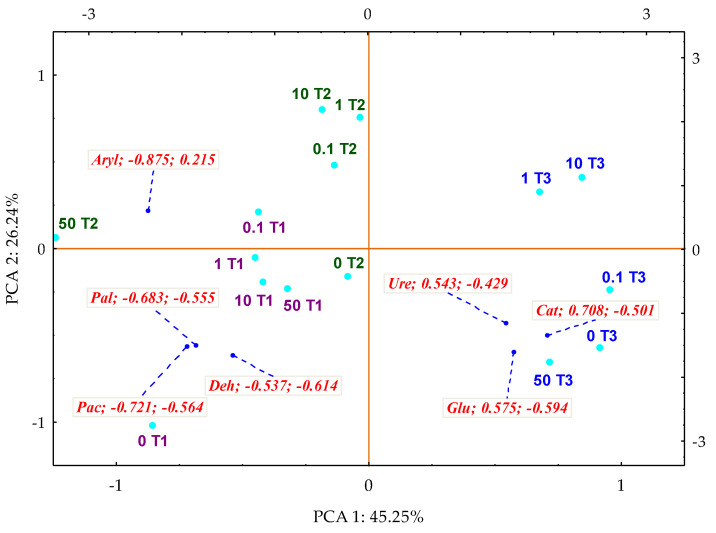
Coefficients of impact (IF_Pc_) of *Perna canaliculus* for the enzyme activity in soil contamination with *o*-cresol—PCA method. The end of the vector of the primary variable: Deh—dehydrogenases; Cat—catalase; Ure—urease; Pal—alkaline phosphatase; Pac—acid phosphatase; Glu—β-glucosidase; Aryl—arylsulfatase; —cases: doses of *o*-cresol mg kg^−1^ DM of soil: 0; 0.1; 1; 10; 50; T1—day 15, T2—day 30, T3—day 45 of the experiment.

The influence of mussel shells was presented by using the biostimulation impact factor IF_Pc_, including the application of multidimensional PCA analysis (Figure 13). The value of the first factor (PCA1), which explains 45% of the total data variance (determined based on the distribution of the standardised vector ends corresponding to the IF_Pc_ values), was shaped by Ure, Glu and Cat (positively correlated with this variable), and Aryl, Pal, Pac and Deh (negatively correlated with this variable). Both the values of coordinates of the cases, and the distances between them, emphasised the effective intensification of the activity of all analysed enzymes by the applied biostimulating substance. The dislocation of cases proved that the compilation of 50 mg *o*-cresol kg^−1^ DM of soil and *Perna canaliculus* on day 15 of the study stimulated the activity of Pal, Pac and Deh. On day 30, it stimulated Aryl, and on day 45 of the experiment it stimulated Ure, Glu and Cat. 

The application to the soil of both 1 and 10 mg *o*-cresol kg^−1^ DM of soil and a biostimulating substance resulted in a comparable escalation of enzyme activity, in parallel intervals, except for day 45 of the study. In this pool of objects, the application of *Perna canaliculus* failed to bring the expected results.

**Figure 14:**
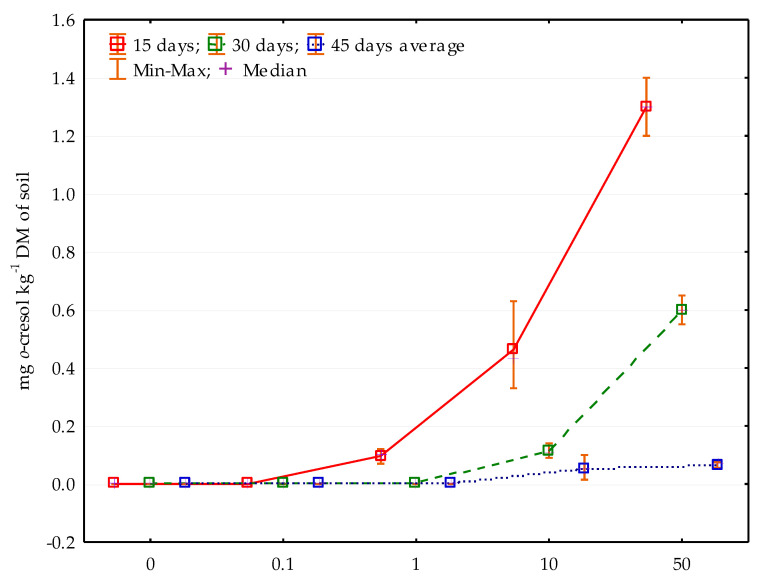
The content of *o*-cresol in the soil after 15, 30 and 45 days of research depending on *o*-cresol contamination; 0; 0.1; 1; 10; 50—doses of *o*-cresol (mg kg^−1^ DM of soil).

For the study to be multi-attribute in nature, the obtained results for the enzymatic activity, which are the criterion of the biological quality of the soil environment, should also be compared with the *o*-cresol residue content in the soil (Figure 14). The content of phenolic compound residues was only noted in soil samples contaminated with 10 and 50 mg *o*-cresol kg^−1^ DM of soil, regardless of the study duration. On day 15 of the experiment, in objects with the highest contamination level (50 mg *o*-cresol kg^−1^ DM of soil), 1.3 mg *o*-cresol was noted, and on days 30 and 45, its amount decreased by 1% and 2%, respectively. Similarly, in pots subjected to the pressure of 10 mg of the studied phenolic compound per kg^−1^ DM of soil, the *o*-cresol content in the soil decreased the most over the initial 15 days (by 96%). 

## 4. Discussion

### 4.1. Counts and Diversity of Bacteria

Changes in the structure of soil microorganisms are indicative of changes in the soil quality defined as the soil’s ability to serve numerous functions, although this indicator is less frequently used in soil assessment systems as a microbiological parameter than as chemical or physical indicators [68,69]. However, there have been reports documenting the inhibitory effect of phenolic compounds on microorganisms [58,70,71]. Our study shows an adverse effect of *o*-cresol on the count of nitrogen-immobilising bacteria and ammonifiying bacteria. This may be because biochemical decomposition of complex phenolic compounds requires both a large number of enzymes and the activation of metabolic pathways, which stimulates the growth of cresol-biodegrading subpopulations and results in absolute changes in the bacterial as well as fungal count [72]. The obtained trends are, however, authenticated by the effect of phenols at a dose of 250 mg dm^−3^ of sewage on the ammonification process, noted at the inhibition level of 63% [73]. Wu et al. [74] also demonstrated the inhibition of phenol degradation by high ammonia concentrations. Interestingly, Wei et al. [75] proved that *Geobacter metallireducens* accumulated nitrates (III) during the process of cresol degradation at a level of 80%. *Pseudomonas* sp. is characterized by a wide range of genes responsible for the degradation of phenolic compounds. In a study by Tian et al. [76], the nucleotide sequence contained the phenol 1,2-dioxygenase gene from *Pseudomonas* sp. PH11, similar to the gene in *Pseudomonas putida* KT24400 and *Pseudomonas arvilla*. *Pseudomonas* sp. also triggers a number of mechanisms involved in the cresol degradation process. Thus, one could expect a stimulation of the abundance of these microorganisms in the presence of *o*-cresol, regardless of the duration of the study.

However, it is necessary to consider the evolution of genes encoding cresol-catabolising enzymes due to environmental contamination with these xenobiotics. The phenomenon of synergism between microbial strains is also important, as reported by Goswami et al. [77]. It manifests itself in the fact that the transformation of cresol in the presence of the *Rhodococcus erythropolis* M1 and *Pseudomonas fluorescens* P1 consortium is much more rapid than in the presence of the pure cultures of these microorganisms, especially with regard to *Pseudomonas fluorescens* P1. However, this does not change the fact that phenolic compounds significantly stimulate the count of *Pseudomonas* sp. [78]. In the presence of *Pseudomonas* sp. 98.5% of cresol was removed and within 40 h, 1200 mg cresol dm^−3^ of sewage was degraded. It was also observed that approximately 70% of the total carbon of this substrate was used to maintain microbiological activity [79]. It is interesting that the highest level of soil contamination with *o*-cresol (50 mg *o*-cresol kg^−1^ DM of soil) caused a significant reduction in the number of fungi in soil subjected to the pressure of this phenolic compound. Atagana et al. [80] noted that this compound degrades much more rapidly than cresol isomers in the meta- and para- positions in the presence of fungi that are sensitive to the presence of *o*-cresol in the soil and represent the *Trichoderma* and *Phanerochaete* genera. However, such research results could be expected, as the toxicity of cresols to fungi is manifested by the peroxidation of cell membrane lipids, which ultimately change the composition of phospholipid fatty acids (PLFAs) [24,81]. Effector molecules (phospholipids) attach to the hydrophobic fragment of 1,2-dioxygenases and, ultimately, to the 3-carboxy-cis, cis-muconate lactonising enzyme [82]. 

The obtained high average values of the CD index of 49.36 and the EP index of 0.824 for organotrophic bacteria prove that they effectively biodegrade *o*-cresols and, thus, belong to the group of r-strategists, which multiply rapidly, irrespective of the degree of disturbance to the ecosystem balance. In contrast, actinobacteria were recognised (based on the CD index) as slowly multiplying or dormant microorganisms, referred to as k-strategists [83]. The obtained low average EP index value of 0.24 for fungi is more intriguing. This trend corresponds to the results obtained by Li et al. [84]. In their study, phenolic acid did not have a favourable effect on the biodiversity of fungi. The obtained result could also be influenced by the phenomenon of reducing the dissociation value of the phenolic compound dissociation (pKa), as well as an increase in the value of the octanol–water partition coefficient for cresol (logP), may cause damage to mitochondria, endoplasmic reticulum and the nucleus [85]. It should also be noted that many contaminants coexist in the natural environment while exerting an antagonistic, additive or synergistic effect on microorganisms [3]. 

However, the preventive measures applied to eliminate the potential inhibitory effect of *o*-cresol produced the expected results. The compilation of the highest level of soil contamination with a phenolic compound (50 mg *o*-cresol kg^−1^ DM of soil) and the biostimulation with *Perna canaliculus* not only resulted in an increase in fungal biodiversity but also significantly intensified the multiplication of nitrogen-immobilising bacteria and ammonifying bacteria. This is understandable, as the New Zealand mussel is a source of peptides, lipids and polysaccharides, the three metabolites significant for both bacterial and fungal growth, which is why its effectiveness was expected [86]. It is also a reliable source of carbon, nitrogen and phosphorus [87].

*o*-Cresol also played a significant role in shaping the soil’s microbiological diversity. According to Delgado-Baquerizo et al. [32], *Alphaproteobacteria* and *Betaproteobacteria* are most often responsible for the abundance of soil phylotypes. Many researchers [88,89] also add to this list the representatives of (mainly) *Proteobacteria, Acidobacteria, Firmicutes* and *Bacteroidetes* phyla. Undoubtedly, as confirmed by the authors’ own study results, these microorganisms also include the representatives of soils contaminated with *o*-cresol, particularly the microbiome represented by bacteria of the *Proteobacteria* and *Firmicute* phyla. These study results were further supported by corresponding observations made by Siczek et al. [71] and Zaborowska et al. [78]. In the authors’ own study, in the soil subjected to *o*-cresol pressure, three genera distinguished by the highest OTU abundance were identified, i.e., *Arthrobacter*, *Devosia* and *Bacillus*. Mahdavianpoura et al. [7] report that microorganisms capable of degrading *p*-cresol in the denitrification process include *Bacillus megaterium, Bacillus aryabhattai* and *Bacillus cereus*. Siczek et al. [71] recognised bacteria of the genus *Bacillus* as significant in phenolic compound biodegradation. In turn, Zaborowska et al. [78], in a study with soil exposed to the effects of phenolic compounds contamination, found a high OTU value for the *Devosia* genus. In the soil samples contaminated with 50 mg *o*-cresol kg^−1^ DM of soil, gram-positive bacteria were dominant. This probably corresponds to the thinner cell wall of gram-negative bacteria and a higher isoelectric point (pH = 4–5) in this group of microorganisms and is due to the weaker bond strength of the gram-negative bacteria in relation to binary toxicants than that of the gram-positive bacteria. Gram-negative bacteria are more vulnerable to cell membrane depolarisation and ATP leakage, which leads to a difference in energy and, consequently, to cell autolysis [90]. However, both the gram-positive bacteria such as *Arthrobacter, Cellulosimicrobium*, *Promicromonospora, Dactylosporangium* and *Geodermatophilus* and those gram-negative, e.g., *Lysobacter, Steroidobacter, Variovorax, Mycoplasma, Caldilinea* and *Sphingopyxis,* are resistant to phenolic compounds [78]. Wu et al. [74] also emphasised the greater resistance and tolerance of aerobic bacteria to this phenolic compound then anaerobic bacteria. A surprising discovery was made by Morrissey et al. [91], who found that phenolic compounds are responsible to a greater extent for the selection of bacterial population than glucose. To assess the microorganisms’ ability to grow under specific conditions, it is also important to consider the metabolism rate constant (*k*). Based on this parameter, a higher biological toxicity of *o*-cresol and *p*-cresol than that of the *m*-cresol isomeride property was determined, which is due to the activation of groups in the ortho- or para-position, which induces a higher reaction activity and biotoxicity in the former phenolic compounds [92]. Among the microorganisms which conduct the dynamic catabolism of phenolic compounds, bacteria of the *Pseudomonas*, *Alcaligenes*, *Nocardia*, *Rhodococcus* and *Acinetobacter* genera, equipped with intradiol dioxygenases that exhibit the quaternary structure composed of at least two subunits with one or two Fe(III) ions per dimer, are also dominant [93,94]. Using the example of *Acinetobacter radioresistens*, it was found that the ionic strength was crucial in the enzyme activity. At a low ionic strength, dioxygenases become the dimer Fe_2_, although they are monomerised after increasing the ionic strength, they continue to retain their catalytic activity, which was not noted for dehydrogenases [92].

### 4.2. Soil Enzymes

In contrast to microorganisms, soil enzymes remain active in the course of the competitive interactions of microorganisms and various carbon sources and are involved in the decomposition of cresols [95]. However, phenolic compounds, including cresols, regulate soil fertility by interfering with the soil enzyme activity [96]. Moreover, Hoostal et al. [97] demonstrated that the complex phenolic structures determine the reaction of extracellular enzymes. The response of dehydrogenases to *o*-cresol pressure in the soil is closely related to the induction of an intensified hydroxylation of inactive ethylbenzene to acetophenone by enzymes, and the ultimate degradation to benzoic acid [98,99]. The ability of dehydrogenase (LOC100783159) to oxidise dihydroacetate (a quinone compound and an aromatic compound derivative) could also be the response to the positive effect of cresol on dehydrogenase activity in the authors’ own study [100]. In a study by Perotti [101], hydroquinone, regarded as a compound toxic to microorganisms, actually increased their count and inhibited the activity of dehydrogenases. Certainly, it is related to the wide range of responses to various phenolic compounds resulted from the presence and position of selected substituents. It is mainly the phenolic methoxy- and hydroxyl-groups and, to a lesser extent, nitro groups, that are responsible for effective inhibition [102,103].

Reports on the toxic effect of quinones on urease activity support the results obtained in the present study. When analysing this enzyme’s response to *o*-cresol, it is necessary to consider that 79–89% of the urease activity in the soil originates from extracellular enzymes stabilised by the adsorption on soil colloids [104]. The kinetics of urease activity inhibition by quinones are related to the cysteine residue’s covalent modification, based on the arylation and oxidation of the quinone thiol groups [105]. According to Zaborska et al. [106], the inhibitory effect of quinones is related to the nucleophilic addition of the Michael-type carbanion to α,*β*-unsaturated carbonyl compounds or, in regard to naphthoquinone, with the redox cycle, which results in the oxidation of thiols [107].

The acid phosphatase response to the contamination of soil with *o*-cresol was more puzzling. The inhibitory effect of the applied phenolic compound can, however, be justified by the fact that phosphatase induces the phosphorylation of disodium phosphate in soils, with this process being a source of toxic quinone [108]. However, consideration should be given to the fact that phenolic compounds decrease the soil pH while selectively activating acid phosphatases by exerting an effect on the enzyme conformation, and the levels of inhibitors, activators and substrates [109]. *β*-Glucosidase on day 45 of the study also appeared to be sensitive to increasing levels of soil contamination with *o*-cresol, which corresponds to the results obtained by Siczek et al. [71]. According to Zhu et al. [3], *o*-cresol is an important carbon and energy source for microorganisms. Therefore, under the pressure of 0.1 mg and 1 mg *o*-cresol kg^−1^ DM of soil, the present study noted a significant increase in the activity of most intracellular enzymes in the soil, irrespective of the duration of the experiment.

Analysing the effect of *Perna canaliculus* as a potential biostimulator of biochemical activity was an important supplement to the assessment of the scale of soil balance disturbances under the pressure of *o*-cresol. *Perna canaliculus* significantly stimulated the activity of individual enzymes at various stages of the experiment, including dehydrogenases, acid phosphatase and alkaline phosphatase on day 15 of the study with spectacular results. The effect was probably generated by the strong correlation between the activity of phosphatases and the availability of nitrogen, whose source was the New Zealand mussel applied into the soil [109]. Similar trends were observed in a study by Zaborowska et al. [58].

Cresols are the main building blocks of the structural nuclei of humic substances, and exhibit a much greater affinity for humic fractions than for fulvic and humic acids. At the same time, hydrogen bonds and the electrostatic and hydrophobic effects are involved in the adsorption process [27]. As the efficient biodegradation of *o*-cresol is guaranteed by the soil pH ranging between 6 and 8, the soil environment temperature at a level of 20–30 °C, and an appropriate degree of soil (aggregation which determines not only the microbiological activity but also the bioavailability of aromatic compounds) one could expect a rapid decomposition of *o*-cresol in the soil under study [110]. In a study by Franchi et al. [111], the sediment with the predominance of the granular fraction was characterised by the highest cresol degradation rate of 11.3 mg dm^−3^ of sediment d^−1^. The oxide-reduction potential value and the ratio between carbon, nitrogen and phosphorus (which amounts to 100:10:1, respectively) also have a significant effect on the process rate [112]. Based on observations by Shibata et al. [113], the cresol degradation time under aerobic conditions was 19 days, while under anaerobic conditions, the half-life period for a phenolic compound ranged from 11–740 days.

## 5. Conclusions

The study found a significant interference of *o*-cresol in the soil microbiome and biochemical activity, and thus could be referred to as a xenobiotic that destroys soil homeostasis. An escalation of the adverse effect of *o*-cresol on the count of microorganisms revealed that the fungi are most sensitive to the presence of *o*-cresol in the soil. In turn, *Pseudomonas* sp. can be considered a microorganism with significant bioremediation potential, with *Arthrobacter* sp., *Devosia* sp. and *Bacillus* sp. representing the *Proteobacteria* and *Firmicutes* phylum bacteria. *Perna canaliculus* is not only an effective biosorbent of organic pollutants; the mussel shells added to the soil at a dose of 5 mg kg^−1^ DM of soil performed the expected function, stimulating both the biochemical and microbiological activity of the soil. In this dose, it proved to be a substance that effectively maintains soil homeostasis. The use of *Perna canaliculus* as a biostimulant is a chance to solve the problem of accumulating waste from mussel shells. Under the pressure of *o*-cresol, the biochemical activity was inhibited. The acid phosphatase, alkaline phosphatase and urease were the most sensitive to the phenolic compound introduced into the soil. The decreasing level of this phenolic compound in the soil had a significant influence on the *o*-cresol toxicity. On day 45 of the research, the amount of phenolic compound applied to the soil decreased by an average of 99.88% ± 0.07. However, it should be noted that the decomposition of *o*-cresol occurred most rapidly within the first 15 days of the experiment.

## Figures and Tables

**Table 1:** Some physico-chemical properties of the soil used in the experiment.

Properties	Unit	Value	Reference
pH_KCl_		7.0	[46]
HAC	mM (+) kg^−1^ DM of soil	6.40	[47]
EBC		165.90	[47]
CEC		172.30	[47]
BS	(%)	96.29	[47]
Corg	g kg^−1^ DM of soil	6.40	[48]
K_e_	mg kg^−1^ DM of soil	180.00	[49]
Ca_e_		2571.40	[49]
Na_e_		20.00	[49]
Mg_e_		59.50	[50]

HAC—hydrolytic acidity, EBC—sum of exchangeable base cations, CEC—cation exchange capacity, BS—base saturation, pH_KCl_—soil reaction, e—exchangeable.

**Table 2:** Chemical identity of *o*-cresol.

Chemical Formula	Chemical Structure	Synonyms	Identification Numbers			
			NIOSH	EPA	RTECS	HSDB
C_7_H_8_O	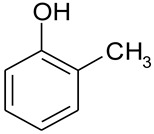	2-methylophenol 2-hydroxytoluene o-cresylic acid	G06300000	F004	U052	1813

NIOSH—National Institute for Occupational Safety and Health; EPA (hazardous waste)—Environmental Protection Agency; RTECS—Registry of Toxic Effects of Chemical Substances; HSDB—Hazardous Substance Data Bank.

**Table 3:** Selected physical and chemical properties of *o*-cresol.

Molecular Weight	MP	BP		WS	PC		VP	BCF
		1 atm	10 mm Hg		Log Octanol/Water	Log K_oc_		
108.14	30.94	191	74.90	25.95	1.95	1.03	0.230	1.25

MP—melting point (°C); BP—boiling point (°C); PC—partition coefficients; WS—water solubility, predicted data (at 25 °C, ppm), VP—vapour pressure, predicted data (at 25 °C, mm Hg); BCF—bioconcentration factor (calculated from K_ow_).

## Data Availability

Data are available by contacting the authors.
